# Plant Chromatin Catches the Sun

**DOI:** 10.3389/fpls.2019.01728

**Published:** 2020-01-24

**Authors:** Clara Bourbousse, Fredy Barneche, Christophe Laloi

**Affiliations:** ^1^Institut de Biologie de l'Ecole Normale Supérieure (IBENS), Ecole Normale Supérieure, CNRS, INSERM, Université PSL, Paris, France; ^2^Aix Marseille Univ, CEA, CNRS, BIAM, Luminy Génétique et Biophysique des Plantes, Marseille, France

**Keywords:** plants, light, photoperception, nucleus, chromatin, 3D genome

## Abstract

Plants use solar radiation as energy source for photosynthesis. They also take advantage of the information provided by the varying properties of sunlight, such as wavelength, orientation, and periodicity, to trigger physiological and developmental adaptations to a changing environment. After more than a century of research efforts in plant photobiology, multiple light signaling pathways converging onto chromatin-based mechanisms have now been identified, which in some instances play critical roles in plant phenotypic plasticity. In addition to locus-specific changes linked to transcription regulation, light signals impact higher-order chromatin organization. Here, we summarize current knowledge on how light can affect the global composition and the spatial distribution of chromatin domains. We introduce emerging questions on the functional links between light signaling and the epigenome, and further discuss how different chromatin regulatory layers may interconnect during plant adaptive responses to light.

## Introduction

From the early studies on daylength and flowering time by Julien Tournois and Georg Albrecht Klebs before the first World War (reviewed in Sage, 1992), a century of research efforts in plant photobiology has permitted the identification of sensory mechanisms that allow plants to cope with fluctuating light conditions over their lifetime (Chory, 2010). Intensity, direction, and spectral composition of light constitute crucial sources of spatio-temporal information for a plant about its environment, for example about photoperiod, season, and presence of neighboring or shade-producing competitors. In addition, while being essential for plant growth and development light can also be harmful at high intensities. Overloading the photosynthetic electron transport chain notably leads to the production of reactive oxygen species (ROS), which can cause irreversible damage to cellular components. Exposure to strong sunlight further exposes plants to the detrimental effects of ultraviolet (UV) radiations on photosynthetic activity, cell integrity, and genome stability. Balancing the needs of photon harvesting for photosynthesis with photoprotection and developmental responses to changing light conditions is therefore at the nexus of plant fitness (Li et al., 2009; Croce and van Amerongen, 2014; Rochaix, 2014; Demarsy et al., 2018).

**Figure 1:**
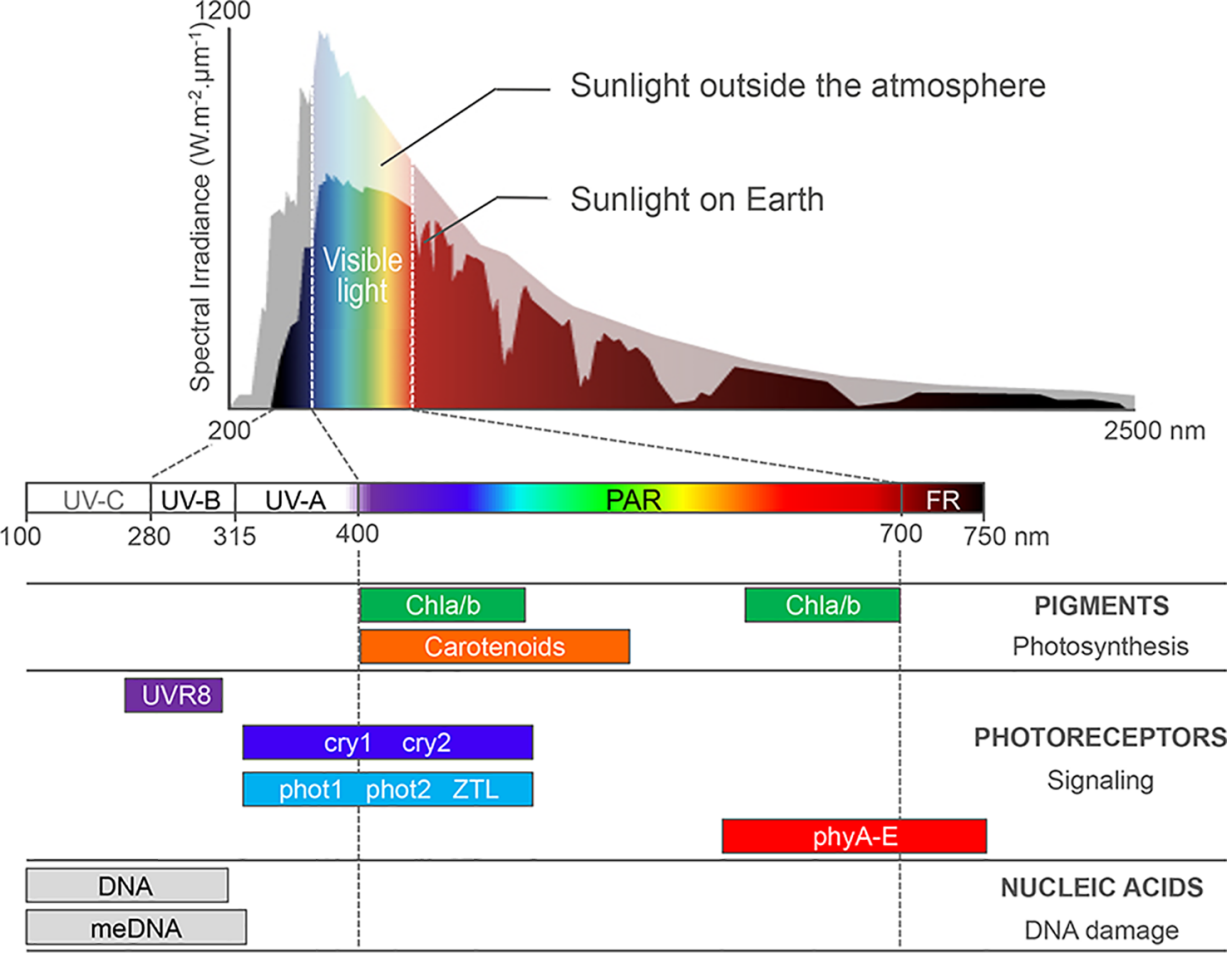
Sunlight absorption by Arabidopsis cell compounds. Most sunlight visible wavelengths penetrate the atmosphere, whereas harmful UV-C and part of UV-B/A are absorbed by ozone in the atmosphere. Photosynthetic pigments, such as the carotenoids and chlorophyll a and b (Chla/b) harvest photons in the 400- to 700-nm waveband, which correspond to photosynthetically active radiations (PAR). A battery of nuclear photoreceptors sensitive to blue, red, far-red, or UV-B light trigger specific adaptations of plant development and metabolism to the local environment. Finally, aromatic DNA bases and methylated cytosines absorb light in the UV waveband, enhancing DNA susceptibility to genotoxic damages and mutations. Adapted from Fondriest Environmental, Inc., (2014) and from Kami et al. (2010).

Plants can integrate light signals through a set of cytosolic or nuclear photoreceptors (**Figure 1**). Photoreceptors allow sensing specific solar wavelengths through an associated chromophore (red and far-red light by phytochromes, blue light by cryptochromes, phototropins, and Zeitlupe) or through tryptophan residues in the case of UV RESISTANCE LOCUS 8 (UVR8) (Reviewed in Galvão and Fankhauser, 2015; Demarsy et al., 2018). When excited by photons, photoreceptors initiate complex regulatory cascades ultimately controlling the expression of vast repertoires of light-responsive genes. In the Arabiopsis model plant species, key signaling components are the photomorphogenesis master repressors DE-ETIOLATED-1 (DET1) and CONSTITUTIVE PHOTOMORPHOGENIC 1 (COP1) proteins that control the stability of photoreceptors and transcription factors, such as the PHYTOCHROME INTERACTING FACTORS (PIFs) and ELONGATED HYPOCOTYL 5 (HY5) (reviewed in Casal, 2013; Galvão and Fankhauser, 2015; Seluzicki et al., 2017). Among these factors, the discovery of DET1 association to nucleosomes has constituted a first molecular function linking light signaling and chromatin (Benvenuto et al., 2002). The precise impact of this evolutionarily conserved factor and of related proteins on chromatin has just started to be unveiled (reviewed in Fonseca and Rubio, 2019). More generally, functional genetic studies have now unveiled the importance of chromatin-level control of gene expression in plant adaptive responses to light conditions. Under high light irradiance, chromatin responses to damaging doses of UV or to stressful plastid activity further contribute to preserving genome activity and stability.

Multiple histone post-translational modifications and chromatin-bound factors act sequentially or in combination to fine-tune the transcriptional activity of light-responsive genes. A first set of genome-wide studies allowed identifying a range of histone marks and nucleosome organization dynamics that contribute to efficient gene expression regulation during Arabidopsis cell adaptation to light (Charron et al., 2009; Bourbousse et al., 2012; Sullivan et al., 2014; Pass et al., 2017). At a larger scale, cytological approaches have identified dynamic changes of chromosomal domains and of specific genes into the nuclear space in response to light signaling. The corresponding pathways converging onto specific chromatin loci or regulating either abundance or activity of chromatin machineries have also started to emerge. Here we report the main advances in understanding the chromatin mechanisms used by plants to cope with solar radiation.

## Light Perception Triggers Massive Nuclear Reorganization

### Heterochromatin Dynamics

**Figure 2:**
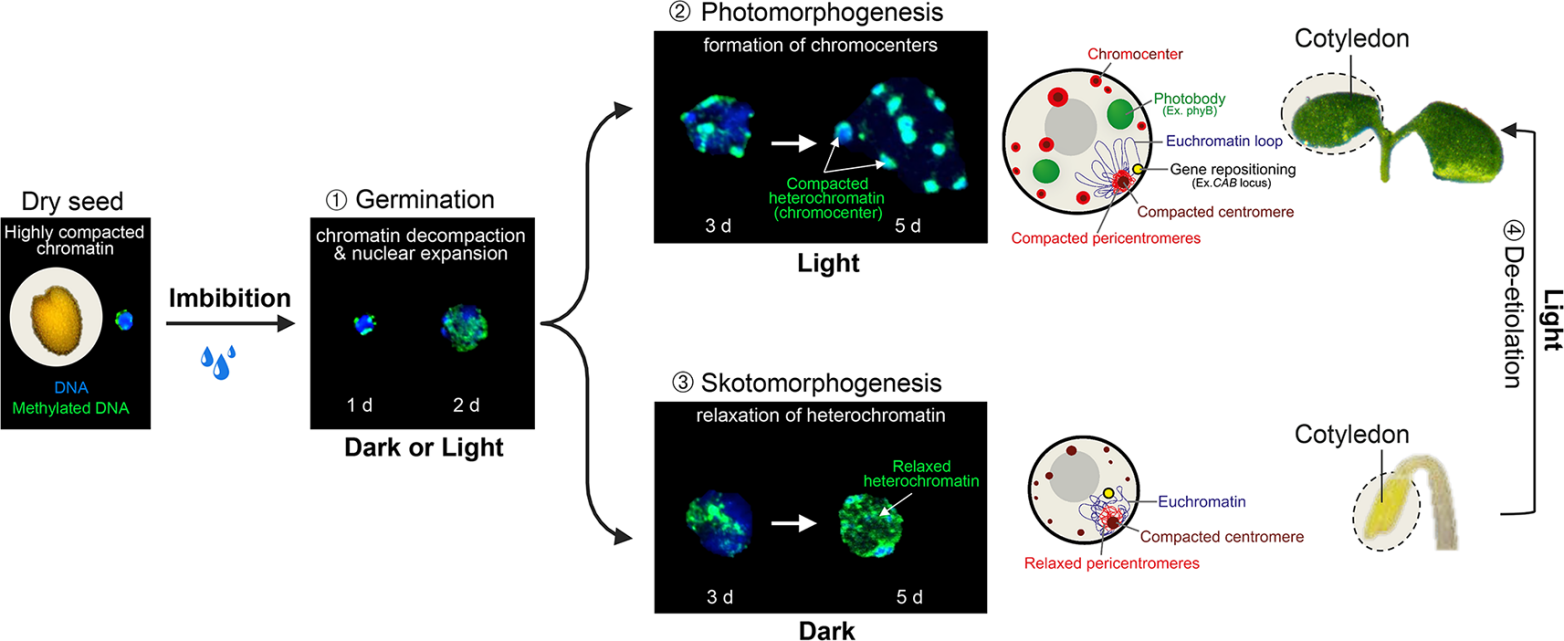
Light-dependent nuclear organization dynamics in cotyledons. During the first 2 days of germination, seed imbibition initiates nuclear expansion and heterochromatin decondensation, as shown by the dispersed signal of methylated DNA immunolabeling in both dark and light conditions (Phase 1). From the third day of germination, light perception triggers further nuclear expansion and the re-compaction of pericentromeric domains within cytologically observable chromocenters (Phase 2), whereas in the absence of light, heterochromatin is further decondensed (Phase 3). Skotomorphogenic nuclear phenotypes can rapidly be converted to a photomorphogenic-type organization during cotyledon de-etiolation (Phase 4). Cotyledon de-etiolation also involves the relocation of light-inducible genes to the nuclear periphery (such as the *CAB* locus), and the aggregation of photoreceptors and downstream signaling components, such as phyB, COP1, PIFs, and TZP into sub-nuclear speckles referred to as photobodies. d; days post-imbibition.

The monitoring of nuclear architecture variations during Arabidopsis post-embryonic cotyledon development has unveiled a serial reorganization process during germination and seedling establishment that relies on light perception (**Figure 2**). In dry seeds, cotyledon nuclei are extremely small while heterochromatic domains, mostly constituted of centromeres, pericentromeres, and silent repeat elements, such as Transposable Elements (TEs) (reviewed in Simon et al., 2015), are highly compacted in two to four subnuclear foci. This peculiar nuclear phenotype may constitute a protective mechanism to desiccation (van Zanten et al., 2011). Upon seed imbibition, a first transition involves an extensive relaxation of heterochromatin and moderate nuclear expansion (**Figure 2**; Phase ➀), which is achieved under either dark or light conditions (Bourbousse et al., 2015). A second step occurring 3 days after imbibition involves additional increase of nuclear size and the re-compaction of dispersed heterochromatin regions into 8 to 10 sub-nuclear regions referred to as chromocenters (Mathieu et al., 2003; Douet et al., 2008; van Zanten et al., 2011), a typical organizational scheme observed in most cell types of Arabidopsis adult plants (Fransz et al., 2002). This second step is dependent on light signaling (Phase ➁) as the formation of conspicuous chromocenters relies on blue light sensing by cryptochromes. In the absence of light, heterochromatin compaction is both inhibited by the DET1 and COP1 signal integrators (Phase ➂). This arrest is rapidly released during de-etiolation (Phase ➃) suggesting that nuclear development is poised prior to the acquisition of phototrophy (Bourbousse et al., 2015).

Upon transition to adult developmental stages, Arabidopsis nuclear architecture also undergoes several reorganizational events driven by light signals (reviewed in van Zanten et al., 2012). This is notably the case in rosette leaf mesophyll cells during the transition from vegetative to reproductive growth. Leaves integrate endogenous and environmental signals to trigger the conversion of apical shoot meristems into flowering meristems. Remarkably, in leaf mesophyll nuclei, most chromocenters undergo a significant disruption a few days before bolting with the dispersion of 5S rDNA and pericentromeric repeats (Tessadori et al., 2007). This transient phenomenon is developmentally controlled, as chromocenters are restored 3 days after bolting. The decompaction process relies on cry2-mediated blue light sensing, which therefore has an opposite effect during this transition than during cotyledon morphogenesis where it triggers chromocenter formation. Flowering time is delayed when *CRY2* function is impaired (Guo et al., 1998), suggesting that a large-scale chromatin decompaction episode is linked to the flowering transition (Tessadori et al., 2007).

A link between light fluence rate and chromatin spatial organization is further suggested by the observation that exposure to low light intensity for a few days triggers the dispersion of most chromocenters in rosette mesophyll cells (Tessadori et al., 2009; van Zanten et al., 2010b; van Zanten et al., 2010a). Again, this event is reversible and relies on light signaling through cry2 and phyB photoreceptors (reviewed in van Zanten et al., 2010a). Interestingly, this mechanistic link might be adaptive in the wild, as population genetics analysis of *Arabidopsis thaliana* accessions identified a correlation between latitude of origin, PHYB amino acid sequence polymorphisms and heterochromatin compaction levels in mesophyll cells (Tessadori et al., 2009; Snoek et al., 2017). Heterochromatin compaction might therefore be an integral part of the plant response to short-term fluctuating light as well as to the annual light radiation regime sensed by phytochrome photoreceptors having evolved different sensitivities to local sunlight conditions.

### Light Regulation of Sub-Nuclear Micro-Environments

In addition to heterochromatin dynamics, Arabidopsis nuclei are subjected to several reorganization events in response to light, which directly impact euchromatic loci, and therefore also protein-coding genes. A landmark study using single-locus cytological experiments unveiled that several light-inducible genes, such as *CAB* (chlorophyll a/b-binding proteins) and *RBCS* (*Rubisco small subunit*), are re-positioned from the interior to the periphery of nuclei during Arabidopsis cotyledon de-etiolation (**Figure 2** Phase ➃). In this context, the physical movement of genes might hypothetically facilitate mRNA export through nuclear pore complexes. Being promoted by phytochrome-dependent red light sensing and inhibited by COP1, DET1, and PIFs, this process appears to be controlled by light signaling (Feng et al., 2014a). Still, as for heterochromatin condensation dynamics, the functional implications of light-dependent gene motion on transcription or on mRNA processing and export remain to be assessed. This could possibly be achieved by artificially tethering a given locus to distinct sub-nuclear compartments and monitoring its resulting transcriptional activity.

Gene repositioning is a dynamic process that echoes the rapid relocalization of light signaling components within conspicuous nuclear speckles, a process observed in both dicotyledonous and monocotyledonous plants when exposed to light. More precisely, upon photoexcitation most nuclear-localized photoreceptors, including the five phytochromes phyA-E, UVR8, cry2, and possibly also cry1, concentrate into nuclear bodies referred to as “photobodies.” The function and composition of the diverse types of photobodies remain elusive. They have been envisioned as either subnuclear sites of light signaling, of protein degradation or as transcription hubs (Van Buskirk et al., 2012), three functions that might be dynamically interconnected. The colocalization of multiple transcriptional regulators such as COP1, PIFs, and TANDEM ZINC-KNUCKLE PLUS3 (TZP) within nuclear microenvironments supports this scenario (reviewed in Van Buskirk et al., 2012; Perrella and Kaiserli, 2016).

### Other Types of Light-Induced Nuclear Reorganization Events

Light perception directly influences nuclear DNA content, a feature with potential high impact on nuclear organization and activity. Low light intensity dampens endoreduplication, for example maintaining low ploidy levels in Arabidopsis leaves (Cookson et al., 2006). A similar trend is at play in etiolated hypocotyls where darkness triggers an additional endocycle (Gendreau et al., 1997) while, conversely, cotyledon de-etiolation engages an increase of DNA ploidy levels (Lopez-Juez et al., 2008). This contrast illustrates the organ-specificity of genome responses to light. DNA content and nuclear volume being positively correlated in angiosperms (Jovtchev et al., 2006), endoreduplication presumably allows for a rapid increase in nucleus and cell size, which is promoted in hypocotyls during seedling etiolation.

There is evidence that ploidy may then influence nuclear organization and gene expression during photomorphogenesis. Firstly, the sub-nuclear positions of chromocenters tend to vary with ploidy level and nuclear volume in Arabidopsis pavement cells, highly endoreduplicated nuclei having more internal chromocenters (Poulet et al., 2017). Second, nuclei with elevated ploidy levels display lowly condensed heterochromatin and low chromatid cohesion (Schubert et al., 2012). Of note, endopolyploidy potentially also influences gene expression by gene dosage effects (Doyle and Coate, 2018). Finally, exposure to high light intensity appears to stimulate endopolyploidy in epidermal pavement cells of Arabidopsis and *Phaseolus* (Kinoshita et al., 2008). Increasing genome copy number may in this case allow to cope better with the mutagenic effects of UV light exposure (Gegas et al., 2014).

Plant responses to damaging solar irradiations constitute another type of light-related process of particular interest, especially if considering not only the deleterious effect of UV on DNA but also the plant capacity to use UV-B perception to modulate photomorphogenesis (Jenkins, 2017; Yin and Ulm, 2017). Despite the presence of sunscreen components and of efficient DNA-repair pathways induced by light, UV radiations have now been shown to impact plant epigenomes in multiple ways (reviewed in Kimura and Sakaguchi, 2006). The role played by chromatin processes in plant DNA repair after UV damage and the mechanisms of chromatin restoration after the completion of repair have been detailed elsewhere (see for example Donà and Mittelsten Scheid, 2015; Molinier, 2017). On a different note, damaging doses of UV-B increase the appearance of heritable DNA mutations with higher frequency on 5-mC cytosines, especially in TC sequence contexts. This highlights the influence of the epigenome on genome integrity (Willing et al., 2016). This effect might relate to the high UV-B absorbance by methylated pyrimidines, which potentially enhances the formation of photoproduct dimers (reviewed in Molinier, 2017). *Vice versa*, UV-C induced photolesions have recently been shown to be a source of DNA methylation changes in heterochromatin (e.g., over pericentromeric TEs) (Graindorge et al., 2019). These findings unveil intricate links between DNA repair factors and the accurate maintenance of epigenome integrity.

UV exposure has further been shown to trigger large-scale heterochromatin rearrangements in Arabidopsis (Graindorge et al., 2019). Still, as for blue-light induced chromocenter relaxation, their functional meaning remains unappreciated. In mammals, heterochromatic regions appear to be particularly prone to DNA mutations, possibly representing a peripheral umbrella to harmful radiation for genes located in the euchromatic interior of the nuclei (Schuster-Böckler and Lehner, 2012; Takata et al., 2013; Smith et al., 2017). Future studies might assess whether different plant heterochromatin organizational patterns or variations in genome topology differ in mutational rates.

## Light Signaling Modulates the Epigenome Landscape

### Light Signaling Controls the Abundance of Chromatin Modifiers and Homeostasis of Histone Marks

**Figure 3:**
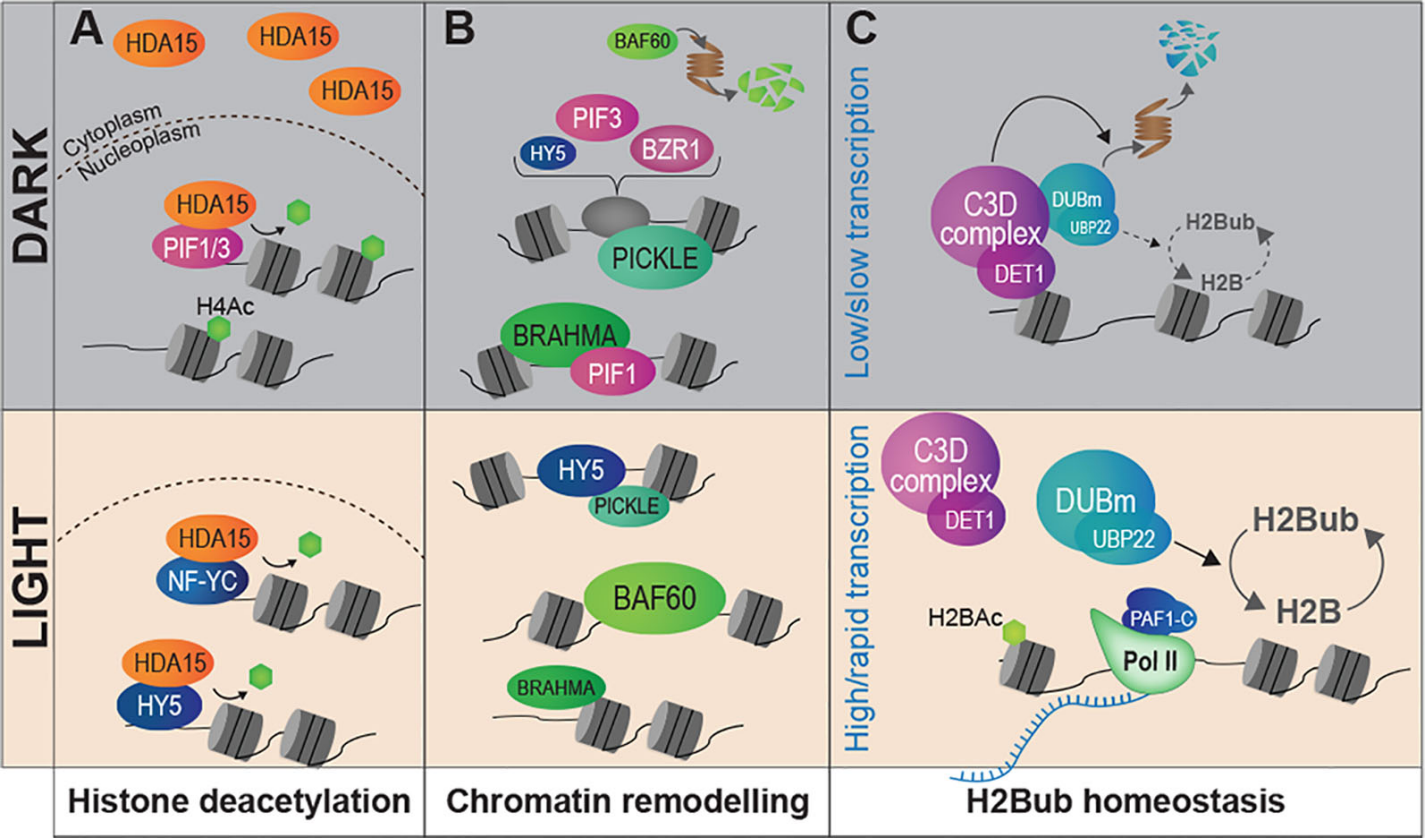
Light signaling controls the abundance of chromatin modifiers and the homeostasis of histone marks. **(A)** Light signaling modulates the sub-cellular partitioning of HDA15, a histone deacetylase that tends to relocate to the nucleus in response to light. In darkness, low amounts of HDA15 retained in the nucleus could target specific light-induced genes through physical association with PIF3. Under light conditions the bulk of HDA15 proteins locate in the nucleus and can associate with NF-YC and HY5. **(B)** The availability of chromatin remodeler subunits is influenced by light conditions. PICKLE and BRAHMA are abundant in darkness whereas BAF60 is expressed and stabilized under light where it may antagonize PIF4 activity. Direct interactions with key TFs of the light-signaling pathway have been demonstrated for PICKLE (with HY5 and PIF3) and for BRAHMA (with PIF1). **(C)** The co-transcriptional H2Bub histone mark is more abundant over most genes in light than dark-grown seedlings. The C3D complex associates to non-acetylated histone H2B and controls the stability of a de-ubiquitination module (DUBm), notably through targeted ubiquitin-mediated proteolytic degradation in darkness. Modulation of H2Bub homeostasis over the epigenome may be related to dampened RNA Pol II activity in cotyledon nuclei in darkness.

Landmark work by the Gray's laboratory allowed linking histone acetylation with nucleosome occupancy and transcription at the *PetE* photosynthetic gene promoter in green and etiolated shoots of pea seedlings (Chua et al., 2001; Chua et al., 2003). From then, an ever increasing number of studies have dissected the role played by chromatin modifying activities in light-regulated gene expression (reviewed in Li et al., 2012; Barneche et al., 2014; Perrella and Kaiserli, 2016; Xiao et al., 2017; Duarte-Aké, 2019). Accordingly, several mutants affected in the deposition or the removal of histone post-translational modifications display photomorphogenesis-related phenotypes. For example, Arabidopsis mutants in the *GCN5* and *TAF1* histone acetyltransferases (HAT) display shorter hypocotyls and lower expression of light-inducible genes, and so, are hyposensitive to light. On the contrary, knocking-out *HD1* or *HDA15* histone deacetylase (HDAC) genes trigger exaggerated inhibition of hypocotyl elongation and other light hypersensitivity phenotypes (Benhamed et al., 2006; Liu et al., 2013). Histone acetyltransferases such as HAF1 and HAC1 are also at play to control gene expression and adaptive developmental responses to UV-B signaling (e.g., plant growth inhibition, flowering time acceleration) (Fina et al., 2017). Hence, photomorphogenesis appears to involve a tight balance between the opposing activities of histone acetyltransferases and deacetylases. In line with these findings, *DET1* hypomorphic mutants display a wide deregulation of histone acetylation levels (Nassrallah et al., 2018). A gene-specific regulatory mechanism involves the light-controlled nucleo-cytoplasmic partitioning of the HDA15 histone deacetylase and its physical association with the transcription factors HY5, PIF3, and NUCLEAR FACTOR-Y C (NF-YCs) in the nucleus (Alinsug et al., 2012; Liu et al., 2013; Tang et al., 2017; Zhao et al., 2019) (**Figure 3A**). Finally, a forward genetic screen aimed at identifying novel regulators of carbon and light signaling identified SDG8, a histone methyltransferase responsible for maintaining high levels of H3K36me3 towards 3′-end of many photosynthetic and metabolic genes (Li et al., 2015).

Other than histone modifiers, the regulation of chromatin functional states in response to light also involves chromatin remodelers. By displacing nucleosomes or facilitating histone exchange, these factors can modulate nucleosome positioning and histone–DNA interactions, thereby influencing local access to transcriptional machineries [reviewed in (Han et al., 2015)]. The importance of such regulatory mechanisms in plant adaptations to light is illustrated by the hundreds of photodynamic DNase/MNase Hypersensitive Sites (DHS) identified in Arabidopsis during seedling de-etiolation or during cell adaptation to light, which typically lie next to light-responsive genes (Sullivan et al., 2014; Pass et al., 2017). A member of the CHD3 family of chromatin remodelers, PICKLE (also referred to as Enhanced Photomorphogenesis 1) has been identified in a forward genetic screen for negative regulators of photomorphogenesis (**Figure 3B**). PICKLE accumulates and associates with the promoter of hypocotyl elongation promoting genes in darkness, thereby preventing deposition of the repressive *Polycomb*-associated histone mark H3K27me3 (Jing et al., 2013). PICKLE interacts with the master regulatory transcription factors HY5, PIF3, and BZR1, which target its chromatin remodeling activity to light-responsive loci (Jing et al., 2013; Zhang et al., 2014). Similarly, two subunits of the SWI/SNF-type ATP-dependent family of chromatin remodelers also show a light-dependent accumulation. Following exposure to light, the accessory subunit BAF60 (also named CHC1 or SWP73B) accumulates and is recruited to the promoters of genes regulating hypocotyl elongation, possibly antagonizing PIF activity through competitive binding onto G-box motifs (Jégu et al., 2017). Reciprocally, abundance of the BRAHMA SWI2/SNF2-type ATPase is lowered upon exposure to light, thereby promoting expression of chlorophyll biosynthetic genes. The PIF1 transcription factor physically associates with BRAHMA, again mediating a *cis*-regulatory gene repression mechanism (Zhang et al., 2017). Taken together, these findings exemplify how light signaling pathways target distinct chromatin remodeling machineries to operate a tight and fine tuning of the light-responsive gene repertoire. They also unveiled how light perception influences the availability of chromatin machineries, a regulatory mechanism potentially mediating large effects over the epigenome.

Correspondingly, bulk levels of histone marks are themselves controlled during cell specification, a critical process in human cancer (Berger, 2000; Jeusset and McManus, 2019) that emerged in plant systems first in the context of gamete formation (Baroux et al., 2007; She et al., 2013; He et al., 2019). For example, abundance of linker histone variant H1.3 and monoubiquitinated histone H2B (H2Bub) is globally regulated in Arabidopsis seedlings in response to light (Rutowicz et al., 2015; Nassrallah et al., 2018). While the function of *H1.3* gene induction by low light remains unclear, establishment of the H2Bub mark is thought to facilitate RNA Polymerase II processivity across nucleosomes, notably through co-transcriptional cycles of histone H2B ubiquitination/de-ubiquitination (Henry et al., 2003). In Arabidopsis, H2Bub deposition is necessary *in cis* for efficient inducibility of hundreds genes during de-etiolation (Bourbousse et al., 2012). Recent work further unveiled that the C3D complex (made of COP10, DET1, DDB1, and DDA1) associates with an H2Bub deubiquitination module (DUBm) that regulates H2Bub levels over most, if not all, Arabidopsis genes (**Figure 3C**). Ubiquitin-mediated proteasomal degradation of this DUBm in response to light appears to allow for a tight control of H2Bub homeostasis (Nassrallah et al., 2018). Low H2Bub abundance over most genes during skotomorphogenesis might hypothetically be linked to the globally low RNA Pol II activity observed in etiolated cotyledons (Bourbousse et al., 2015). These studies echo the recent report of low Polymerase-Associated-1 (PAF1) complex subunits expression and slow RNA Pol II elongation in dark-adapted Arabidopsis plants (Godoy Herz et al., 2019).

In brief, besides gene-specific targeting mechanisms and spatial rearrangements of chromatin domains, light appears to modulate the homeostasis of several histone marks, notably through a tight control of chromatin modifiers' availability. Light therefore not only reshuffles chromatin states at induced and repressed genes but also deeply modify chromatin composition as a whole. Such genome-level chromatin changes might enable an adjustment of the chromatin landscape to the cell transcriptional status (**Figure 3C**).

### Direct Paths From Light Signals to Chromatin States

As presented in the previous sections, chromatin modifiers and remodelers can be targeted to specific loci by the means of light-controlled transcription factors thereby constituting direct signaling pathways. Noteworthy, phytochromes also interact with PIFs (Ni et al., 1998; Leivar and Quail, 2011), cryptochromes with CIB1 and PIF4 (Liu et al., 2008; Ma et al., 2016; Pedmale et al., 2016) and UVR8 can interact with BES1 (BRI1-EMS-SUPPRESSOR1) and BIM1 (BES1-INTERACTING MYC-LIKE 1) (Liang et al., 2018). Profiling of the PHYA, PHYB, CRY1, and CRY2 association to chromatin further shed light on the genomic distribution of photoreceptors in seedlings exposed to either white, blue, far-red, or low blue-light conditions, respectively (Chen et al., 2014; Jung et al., 2016; Ma et al., 2016; Pedmale et al., 2016). The reported binding sites largely overlap with those of light-regulated transcription factors suggesting that photoreceptors bind chromatin indirectly *via* protein-protein interactions. Yet, following the first cytological observations of Arabidopsis CRY2 association to mitotic chromosomes (Cutler et al., 2000), this protein has been reported to display intrinsic DNA-binding properties (Yang et al., 2018). Finally, based on its sequence similarity to human RCC1 (REGULATOR OF CHROMATIN CONDENSATION 1) and on nucleosome binding assays, the UV-B photoreceptor UVR8 has long been hypothesized to act directly on chromatin (Brown et al., 2005; Cloix and Jenkins, 2008; Favory et al., 2009; Binkert et al., 2016). The functional meanings of UVR8 *in vitro* association to chromatin findings remain to be established. These physical associations imply the existence of extremely short paths linking light sensing and molecular implementation in the epigenome.

In addition to photoreceptors, other nuclear light signaling components have the capacity to associate with histones and can therefore be considered themselves as chromatin factors. Noteworthy, DET1 displays high affinity for non-acetylated histone H2B both *in vitro* and *in vivo* and therefore potentially associates to poorly transcribing genes (Benvenuto et al., 2002). Accordingly, Arabidopsis DET1 triggers efficient transcription inhibition when targeted to reporter genes in plants or in a yeast heterologous system (Maxwell et al., 2003; Lau et al., 2011). Still, it remains unclear whether DET1 repressive activity relies directly on targeted degradation of the H2Bub DUBm at regulated chromatin loci *in cis* (Nassrallah et al., 2018) and whether that process is targeted by DET1-associated transcription factors such as HY5 and PIFs (Osterlund et al., 2000; Dong et al., 2014).

Being located close to chromatin, photo-excited photoreceptors or downstream light signaling components therefore have the potential to couple light sensing with a functional rewiring of chromatin *in situ*. Such a direct coupling between light sensing and chromatin may allow for rapid transcriptional reprogramming, thereby resembling metazoan nuclear receptors that combine transcription factor and ligand-binding activities (Perissi and Rosenfeld, 2005). Additionally, the lack of a signal amplification cascade may prevent spontaneous activations that are usually avoided by negative feedback regulations (Kiel et al., 2010).

### Retrograde and Metabolic Signals to Chromatin?

Besides the direct perception of light by nuclear photoreceptors, photosynthetic light harvesting might also impact plant nuclear organization and chromatin states through more indirect paths. Photon harvesting by photosystem antennae directly influences the energetic and redox status of plant cells (Eberhard et al., 2008). Plastids can therefore act as environmental sensors and communicate information about ambient light conditions to the nucleus, notably through retrograde signaling mediated by metabolite and protein signals (Chan et al., 2016). The extent to which retrograde signaling impacts on epigenome organization and function remains largely unexplored. Still, identification of a nuclear topoisomerase VI (Topo VI; Yin et al., 2002) as being essential for the activation of nuclear genes in response to a plastid-derived photo-oxidative stress exemplifies the importance of chromatin-level control of nuclear responses to high light (Šimková et al., 2012). Loss of Topo VI subunits leads to heterochromatin disorganization (Kirik et al., 2007), as observed under dark and low light conditions. In addition, several chromatin modifying enzymes catalytic activity directly relies on the availability of substrate metabolites, such as acetyl-CoA, S-adenosyl methionine (SAM) and adenosine tri-phosphate for histone acetyltransferases, histone/DNA methyltransferases and chromatin remodeling ATPases, respectively. In mammals, direct coupling between redox homeostasis and chromatin state involves the histone deacetylases from the Sirtuin family (reviewed in Suganuma and Workman, 2018), whose activity requires NAD+ as a cofactor. In plants as well, an Arabidopsis Sirtuin-like protein, AtSIRT1, has recently been involved in stress tolerance and metabolic regulations (Liu et al., 2017a). Further investigation is needed to identify how metabolic and redox cellular status impact on chromatin organization and function. In particular, much remains to be explored on the influence of plastid biogenesis and photosynthesis on the chromatin landscape during cell adaptations to environmental changes.

## Linking Variations in Local Chromatin Status With 3D Re-Organization Events

Temporal correlations between light-induced gene motion and chromocenter formation suggested that genome 3D reorganization is an integral part of Arabidopsis de-etiolation (Barneche et al., 2014; Kaiserli et al., 2018). Gene re-positioning or photobody formation could be facilitated when chromatin is globally decompacted. In this scenario, a relaxed chromatin status might retain high flexibility to specify different genome topologies in response to environmental conditions that plant may predictably face. Accordingly, heterochromatin decompaction is an evolutionarily conserved feature common to undifferentiated eukaryotic cells, as shown for mammalian embryonic stem cells or plant protoplasts (Probst and Almouzni, 2011). Dynamic control of the chromatin association of light signaling components might not only allow for targeted gene regulation but potentially also modulate nuclear organization.

As described above, many light signaling components and transcription factors relocate within photobodies upon photoreceptors excitation. Concentrating light transcriptional regulators within a small number of nuclear micro-domains might functionally organize genes that are similarly regulated by light but distantly encoded in the genome. In the absence of clear evidence for “transcription factory” foci in plant nuclei, determining whether photobodies actually correspond to transcriptional foci and whether they contain light-regulated genes might help better understanding the spatial regulation of transcription in plant systems.

Still, although higher-order heterochromatin dynamics usually coincide with genome expression reprogramming events, we lack information on the extent to which chromocenter dispersion in the nucleoplasm actually relies, at the molecular level, on physical changes in the structure or composition of heterochromatic domains. We also do not know how heterochromatin relaxation events functionally impact on euchromatic domains and in particular on protein-coding genes (Feng et al., 2014b; Liu et al., 2017b). Generating high-resolution maps of genome topological variations in response to changing light regimes therefore represents a critical step to characterize gene motion in cell types with distinct light-response specificities. The implementation of chromosomal conformation capture derived approaches (3C; Bickmore and van Steensel, 2013), super-resolution microscopy or adapted chromatin profiling techniques (Re-ChIP, ChIA-PET, etc) applied to peripheral components as in (Bi et al., 2017) or to specific nuclear regions will surely boost these research efforts in the coming years.

## Perspectives

Recent findings unveil that cell differentiation during many plant adaptive responses to light involves large and rapid rearrangements of the epigenome landscape and of genome topology in photosynthetic cells. Still, the functional impact of chromocenter formation, photobody dynamics, and gene re-positioning on transcription remain speculative in most instances. It is also currently unclear whether heterochromatin reorganization and gene motion are mechanistically or functionally interconnected. Regarding Arabidopsis de-etiolation case studies, it remains to be assessed whether nuclear architectural adaptations occurring upon the initial seedling adaptation to light are linked to the dramatic changes in metabolic activity following chloroplast biogenesis, to cell cycle control, to the process of cell differentiation itself or more directly to the cell transcriptional regime. *Vice versa*, chromatin architecture dynamics may also impact photon transmission through plant cell nuclei. An inverted nuclear organization with heterochromatin being concentrated toward the interior of the nucleus indeed constitutes a crucial adaptation of rod cells for nocturnal lifestyle, thereby allowing a more efficient channeling of light (Solovei et al., 2009; Solovei et al., 2013). Whether light-regulated heterochromatin rearrangements can allow for biophysical adaptations favoring photon penetration into certain plant cell layers is largely unexplored. Finally, the potential capacity of light to trigger long-term somatic adaptive responses though priming mechanisms (*transcriptional memory*) or through trans-generational inheritance of UV-regulated epigenetic processes remain poorly investigated as compared to other plant stresses (Müller-Xing et al., 2014).

## Author Contributions

All authors contributed to writing and funding research activity.

## Funding

This work is supported by the EPIPLANT Groupement de Recherche (France). Work by CB and FB is supported by the Velux Foundation (Switzerland), MEMOLIFE (ANR-10-LABX-54), and PSL* Research University (ANR-11-IDEX-0001-02). Work by FB is supported by the French National Research Agency grants Chromalight (ANR-18-CE13-0004-01) and RiboStress (ANR-17-CE12-0026-02), and the COST Action CA16212 INDEPTH (E.U.). Work by CL is supported by the French National Research Agency (ANR-14-CE02-0010).

## Conflict of Interest

The authors declare that the research was conducted in the absence of any commercial or financial relationships that could be construed as a potential conflict of interest.
